# Reconstructing asynchrony for mechanical ventilation using a hysteresis loop virtual patient model

**DOI:** 10.1186/s12938-022-00986-9

**Published:** 2022-03-07

**Authors:** Cong Zhou, J. Geoffrey Chase, Qianhui Sun, Jennifer Knopp, Merryn H. Tawhai, Thomas Desaive, Knut Möller, Geoffrey M. Shaw, Yeong Shiong Chiew, Balazs Benyo

**Affiliations:** 1 School of Civil Aviation & Yangtze River Delta Research Institute, Northwestern Polytechnical University, Xian, China; 2 Dept of Mechanical Engineering, Centre for Bio-Engineering, University of Canterbury, Christchurch, New Zealand; 3 Auckland Bioengineering Institute, The University of Auckland, Auckland, New Zealand; 4 GIGA-In Silico Medicine, Institute of Physics, University of Liege, Liege, Belgium; 5 Institute for Technical Medicine, Furtwangen University, Villingen-Schwenningen, Germany; 6 Dept of Intensive Care, Christchurch Hospital, Christchurch, New Zealand; 7 School of Engineering, Monash University, Subang Jaya, Malaysia; 8 Dept of Control Engineering and Information Technology, Budapest University of Technology and Economics, Budapest, Hungary

**Keywords:** Asynchrony, Mechanical ventilation, Hysteretic lung mechanics, Hysteresis loop model, Virtual patient, Lung mechanics

## Abstract

**Background:**

Patient-specific lung mechanics during mechanical ventilation (MV) can be identified from measured waveforms of fully ventilated, sedated patients. However, asynchrony due to spontaneous breathing (SB) effort can be common, altering these waveforms and reducing the accuracy of identified, model-based, and patient-specific lung mechanics.

**Methods:**

Changes in patient-specific lung elastance over a pressure–volume (PV) loop, identified using hysteresis loop analysis (HLA), are used to detect the occurrence of asynchrony and identify its type and pattern. The identified HLA parameters are then combined with a nonlinear mechanics hysteresis loop model (HLM) to extract and reconstruct ventilated waveforms unaffected by asynchronous breaths. Asynchrony magnitude can then be quantified using an energy-dissipation metric, *E*_*asyn*_, comparing PV loop area between model-reconstructed and original, altered asynchronous breathing cycles. Performance is evaluated using both test-lung experimental data with a known ground truth and clinical data from four patients with varying levels of asynchrony.

**Results:**

Root mean square errors for reconstructed PV loops are within 5% for test-lung experimental data, and 10% for over 90% of clinical data. *E*_*asyn*_ clearly matches known asynchrony magnitude for experimental data with RMS errors < 4.1%. Clinical data performance shows 57% breaths having *E*_*asyn*_ > 50% for Patient 1 and 13% for Patient 2. Patient 3 only presents 20% breaths with *E*_*asyn*_ > 10%. Patient 4 has *E*_*asyn*_ = 0 for 96% breaths showing accuracy in a case without asynchrony.

**Conclusions:**

Experimental test-lung validation demonstrates the method’s reconstruction accuracy and generality in controlled scenarios. Clinical validation matches direct observations of asynchrony in incidence and quantifies magnitude, including cases without asynchrony, validating its robustness and potential efficacy as a clinical real-time asynchrony monitoring tool.

## Background

Mechanical ventilation (MV) is a core therapy for respiratory failure patients in the intensive care unit (ICU) [1], and is particularly important for managing Covid-19 patients [2, 3]. Model-based methods have proven potential and accuracy [4–8] in predicting patient-specific response to care, and thus for guiding and optimizing MV care. In particular, to avoid ventilator induced lung injury (VILI) and thus reduce length of stay, mortality, and cost [9]. However, significant inter- and intra-patient variabilities in lung mechanics and condition can make model identification difficult reducing the accuracy of lung mechanics identified [8]. This issue is compounded when patients exhibit spontaneous breathing (SB) efforts, or any mismatch with the ventilator delivery, more generally referred to asynchrony [10–13].

Patient SB effort is common as completed paralysis and heavy sedation of MV support may lead to ventilator induced diaphragmatic dysfunction [14]. Thus, patient–ventilator interaction is frequent in long-term MV treatment with respiratory work done by both the ventilator and patient. Patient-ventilator asynchrony is commonly seen when patient effort exists while the patient–ventilator interaction is not optimal. Therefore, patient–ventilator asynchrony is defined as a mismatch between the patient, regarding time, flow, volume, or pressure demands of the patient respiratory system, and the ventilator, which supplies such demands during MV [15]. In contrast, patient–ventilator synchrony is ventilator setting gas delivery matched patient respiratory demand. Clinical data demonstrate patient SB effort can cause up to 85% asynchrony rate [15], associated with failure of MV weaning and longer length of stay [16]. Therefore, it is important to extract the true underlying ventilated lung mechanics response from asynchronous measured pressure and flow waveforms to best estimate the incidence and magnitude of asynchrony, and optimize MV settings to mitigate this effect and optimize care.

Clinically, visual inspection of ventilator waveform (pressure and/or flow) has been a major approach for bedside detection of patient asynchrony, while less than 25% of health professionals in ICU were able to identify asynchronies with good accuracy [17, 18]. Computer algorithms have been considered a useful tool to overcome the professional bias. However, current researches are largely focused on the detection of asynchrony occurrence and identification of asynchrony types [16, 19–23], while few of them are able to identify the magnitude of asynchrony or reconstruct the distorted ventilated waveforms.

In particular, reconstruction of asynchronous breath is a challenging task as the patient- and breath-specific responses to MV are changing over time. Thus, it is an identification of an unknown, unpredictable, and unmodeled input to model-based methods altering their accuracy. Reconstruction of unaltered waveforms in asynchrony has been studied using a well-validated single compartment linear lung model [24–28]. However, these methods require a multistep analysis or iteration of the pressure waveform with accuracy and robustness depending on the convergence of the algorithm. Moreover, reconstruction using only one-dimension pressure waveforms might lead to unidentifiability issue due to incomplete information of the couple effect. Therefore, it is needed to develop a more robust algorithm to consider the coupled effect of both pressure and volume without suffering convergence issues.

There are a range of asynchrony types, influenced by neural inspiratory time and ventilator settings among other factors [11, 16, 26, 29, 30]. It is noted that the factors affecting asynchrony are substantial [31], while synchronization requires reconstruction to obtain the magnitude of asynchrony and the patient-specific lung mechanics to optimize the MV setting. Machine-learning methods and other algorithms have been applied to identifying, but not reconstructing, asynchronous breaths from measured waveforms [16, 21, 32, 33]. In particular, automated classification methods or machine-learning methods have been developed to detect ineffective triggering, commonly caused by auto-PEEP or airflow obstruction [19, 20], or double triggering due to longer inspiratory time [23]. However, these methods require pre-annotation of the training sets, which is not practical and did not generalize well when a number of different types of asynchrony types were presented.

In addition, label training data for classification may be conducted via visual inspection of clinicians, while labels of continuous physical parameters for reconstruction are problematic and inaccurate via human input as the parameter value is not directly available from the reading of waveforms. Thus, these methods mainly focus on the detection of asynchrony, rather than the quantification of asynchrony magnitude and reconstruction. More importantly, these identification methods and the fewer number of automated modeling reconstruction methods [24–28] do not yet provide realistic or consistent reconstruction beyond identifying asynchrony incidence, which is critical to optimizing and personalizing MV care to the broadest number of patients [34–37]. Finally, they are black-box methods, which are unable to provide estimates of lung mechanics critical for clinical interpretation of MV settings, where the main advantage of model-based methods is their enabling of automated monitoring and analysis of MV and lung mechanics [7, 24–28].

In contrast, a recently developed virtual patient model based on hysteretic pressure–volume (PV) loop analysis and hysteresis loop model (HLM) offers more complete respiratory information from measured pressure and volume/flow waveforms. Improved and more robust identification of model-based lung mechanics with a nonlinear mechanics model provides a foundation to identify asynchrony as an unmodeled deviation from expected synchronized breathing response in MV [7]. Its detailed nonlinear mechanics offers the opportunity to thus identify the incidence, type, and magnitude, of asynchrony via identification of each part of the PV loop and subsequent model-based reconstruction.

In addition, hysteresis loop analysis (HLA) has been developed to specifically identify the shape and fundamental mechanics of hysteresis loop for engineering dynamics systems, while the PV loop observed in lung mechanics has found to be equivalent to hysteresis loop [7]. Thus, the change of shape in PV loop caused by patient asynchrony is considered to be equivalent to the change of hysteresis loop due to damage in engineering structures, indicating the feasibility of modeling and predicting lung mechanics using HLA and hysteretic mechanics model. Therefore, the HLA is employed to investigate the underlying mechanics of PV loop for the detection and reconstruction of asynchronous breaths based on its validated capability of identifying hysteretic systems in the prior work [38].

This paper develops a model-based method to identify the incidence and type of asynchrony, as well as their magnitude, using the validated HLM model. The major goal is to provide a direct breath-to-breath estimation of asynchrony incidence and magnitude using only ventilated breath waveform data and a proven virtual patient model framework for real-time bedside monitoring. A unique validation including test-lung experimental data, where asynchronous inputs are known, as well as clinical data are used to validate the method and quantify its performance.

## Results

### Experimental test-lung results and validation

**Fig. 1:**
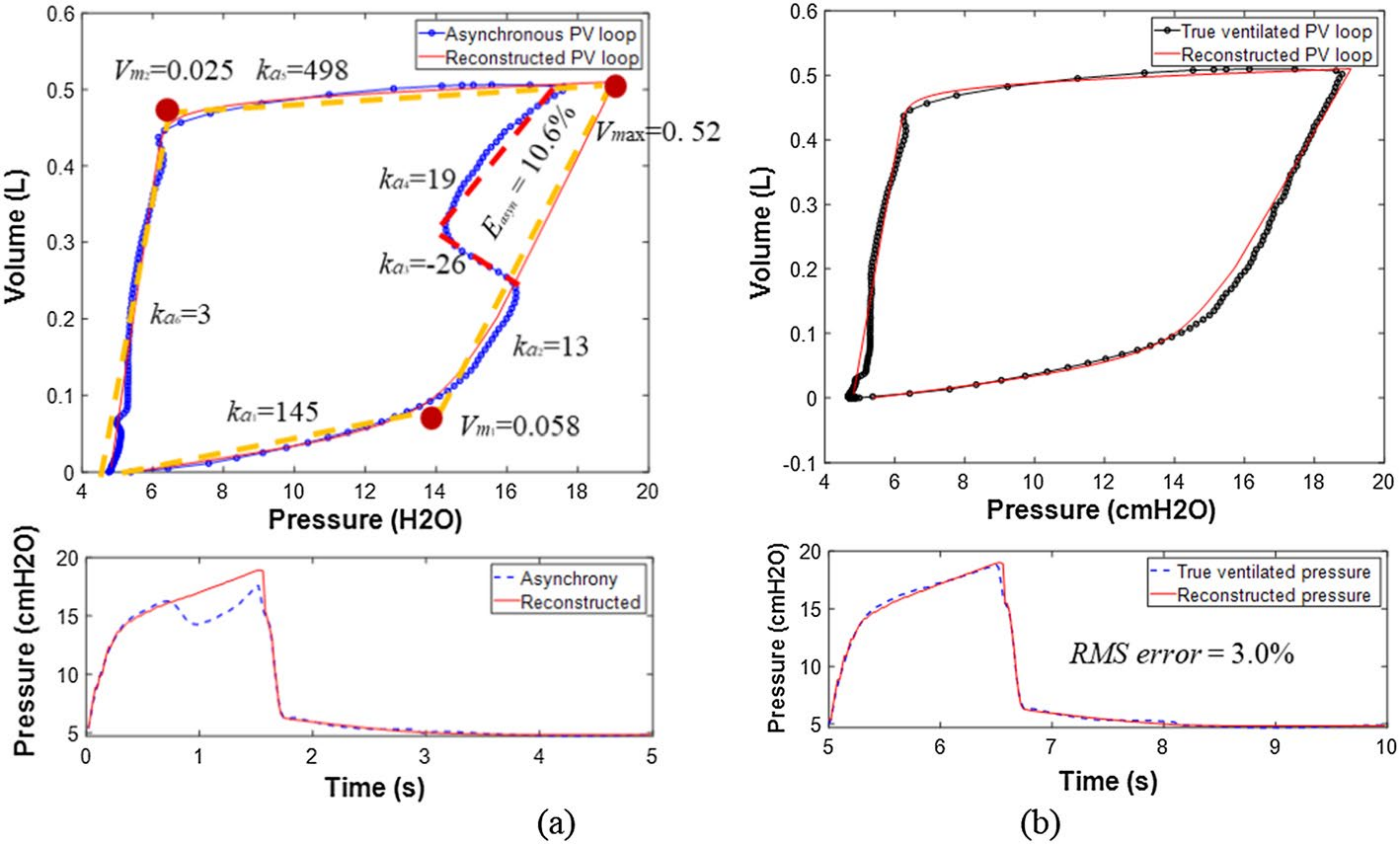
Reconstruction of asynchronous PV loop using HLM (left, **a**) to compare with the true ventilated PV loop (right, **b**)

Figure 1 shows a reverse-triggering example result with comparison of the reconstruction to an unaltered breath in the experimental setup. The reconstructed loop and pressure waveform match the designed non-asynchronous breath very well with root mean squared (RMS) error of 3.0%, indicating successful reconstruction for this example using the proposed method. Calculated *E*_*asyn*_, quantifying asynchrony magnitude, is 10.6%, indicating a significant asynchrony magnitude of 10.6% of the total ventilator supplied work of breathing for an unaltered breath, matching qualitative observation of Fig. 1a.

Table 1 summarizes the reconstruction accuracy for 10 asynchronous breaths under each of the three simulated scenarios. The maximum RMS errors of identification for each scenarios are 3.5–4.1% and the average errors for each scenario are within 3.0–3.2%, a very small range with a tight standard deviation between 0.3 and 0.4%, indicating good robustness and accurate identification over all simulated scenarios.

**Table 1:** RMS errors of reconstruction for experimentally simulated asynchrony for *N* = 10 breaths and each type of asynchrony

Scenario		RMS error (%)											
		1	2	3	4	5	6	7	8	9	10	Mean	SD
1	Reverse triggering	3.0	3.1	3.2	3.0	3.1	3.0	**4.1**	2.8	3.0	3.2	3.2	0.3
2	Premature cycling	3.4	**3.9**	3.0	3.6	3.5	3.1	3.3	3.7	3.3	3.4	3.4	0.3
3	Double triggering	**3.5**	2.6	2.7	2.6	3.3	2.6	2.8	3.5	3.0	3.3	3.0	0.4

**Fig. 2:**
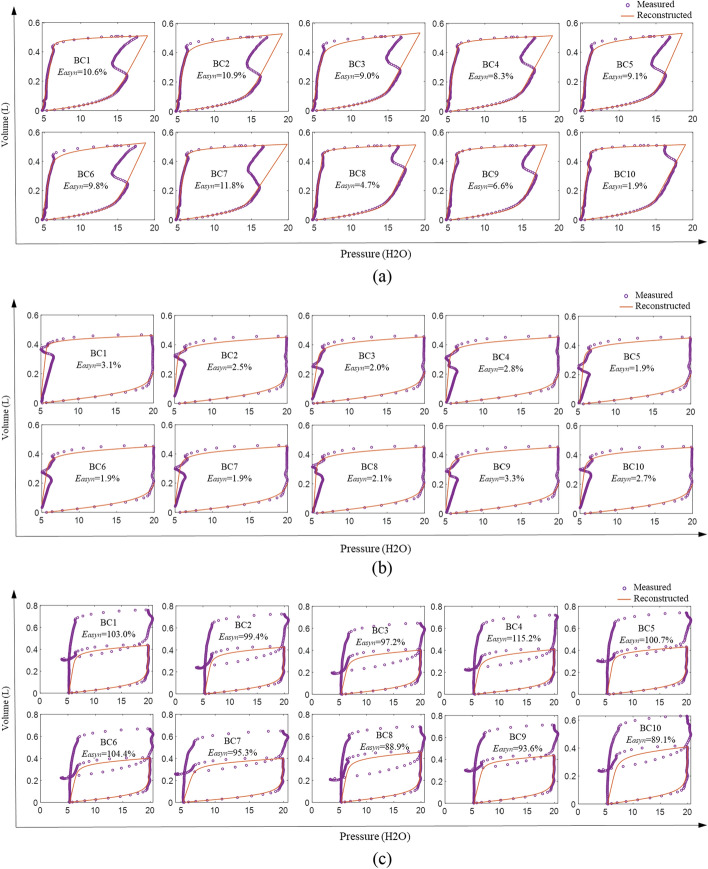
Evaluation of asynchrony effect using *E*_*asyn*_ for types of **a** reverse triggering, **b** premature cycling, and **c** double triggering

Figure 2 shows these results graphically. Reverse triggering led to a range of *E*_*asyn*_ values from the smallest SB effort of 1.9% (BC10) to the strongest SB effort 11.8% (BC7), matching the observed difference of enclosed area between the asynchronous and reconstructed PV loop. Similarly, *E*_*asyn*_ values are small for the premature cycling in Fig. 2b when the SB effort is smaller than the trigger threshold, while the *E*_*asyn*_ values are much higher (> 88%) for the double-triggering breaths seen in Fig. 2c as the SB created negative pressure exceeds the trigger threshold leading to a second breath. The overall results show the ability of the method to reconstruct and evaluate SB during both early (inspiration) and late (expiration) asynchrony using complete information of a breath in a nonlinear, clinically validated model. Hence, it is generalizable to different types of asynchronies, as shown in this test-lung validation.

Finally, the estimation of *E*_*asyn*_ can be readily conducted breath-to-breath automatically for a real-time assessment of asynchrony effect, and there is no need beyond validation to compare to ventilated/paralyzed breaths, as in Fig. 1. It should be noted the HLM virtual patient model and methods are a fully automated process [7]. Finally, the test-lung experimental data enable a simulation of fully ventilated response, thus providing a ground truth for validation, while the availability of a fully paralyzed breath may not be guaranteed for clinical data depending on the patient condition.

### Clinical data results and proof of concept

**Fig. 3:**
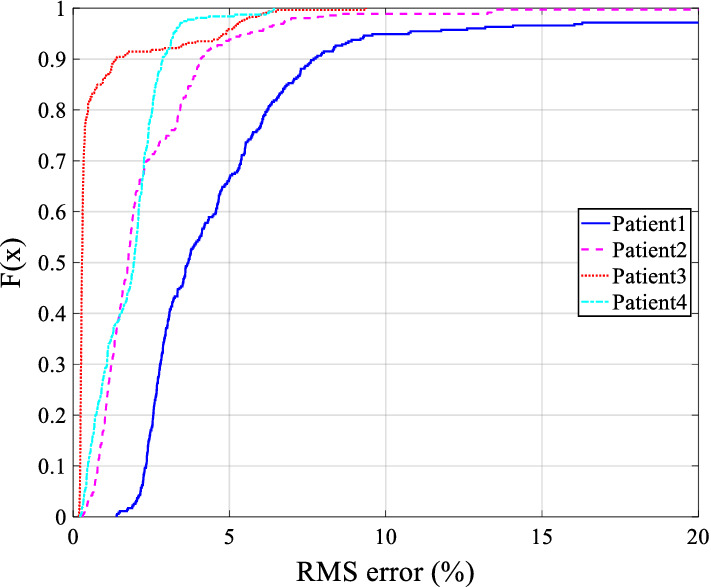
Empirical cumulative distribution (CDF) of reconstruction error for clinical data. F(x) in the y-axis is the CDF of *X* = RMS error (%) in the x-axis

Figure 3 shows the cumulative distribution function (CDF) plot for the RMS reconstruction errors of all breathing cycles for all four patients. Reconstruction errors for each patient are within 10% for 90% of breaths, indicating a good and robust reconstruction accuracy using the proposed method in the presence of noise, comparison across breaths, and real clinical variability.

**Fig. 4:**
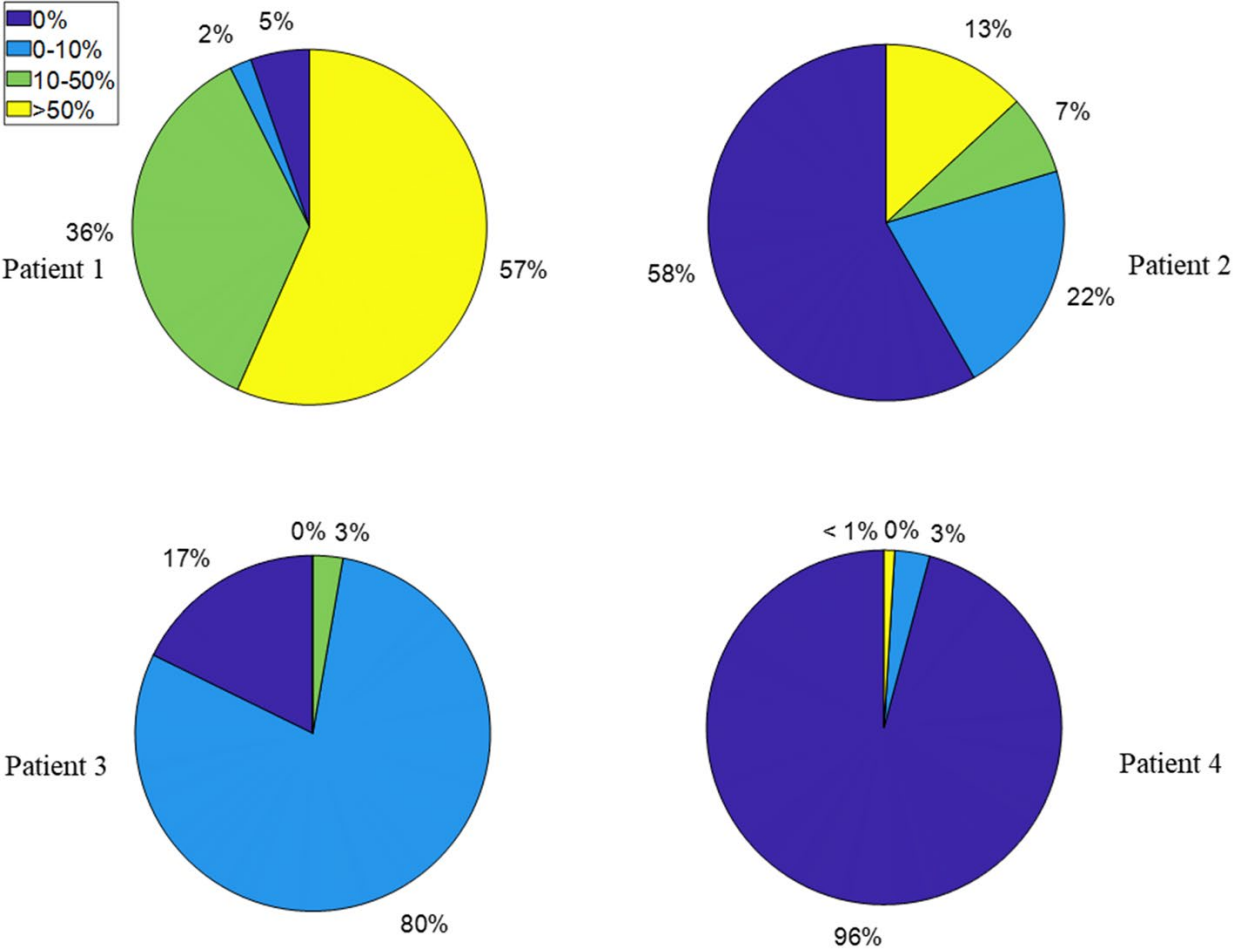
Identified distribution of *E*_*asyn*_ for the 4 patients

The distribution of *E*_*asyn*_ is also quantified in the pie plots of Fig. 4, where 0% error is a normal unaffected breath with no asynchrony. Clearly, Patient 1 has significant asynchrony with 57% breaths having *E*_*asyn*_ > 50%, matching direct manual observations of strong reverse triggering over these breathing cycles. Patient 2 shows 13% of breaths with *E*_*asyn*_ > 50% mainly due to double-triggering effect, where premature cycling was more likely to lead to smaller values (0–10%) of *E*_*asyn*_ for 22% of breaths similarly seen in the simulated data. In contrast, Patient 3 only presents minor reverse triggering, having 80% breaths with *E*_*asyn*_ (0–10%). Finally, no significant asynchrony is identified for Patient 4 with *E*_*asyn*_ = 0 for 96% breaths. There is thus a clear and wide level of inter-patient variability.

## Discussion

A validated virtual patient model for modeling fully sedated MV patient response is extended for the identification, reconstruction, and quantification of asynchronous breathing cycles. Direct tests using clinical data can lack evidence of accuracy, as there are no known exact answers, where the test-lung experimental data provide a comparable, known ground truth for proof-of-concept validation. Thus, the typical asynchrony patterns observed in the clinical data were replicated and simulated based on test-lung experiments. Figures 10 and 12 show very similar asynchronous PV loops between the clinical and simulated data, validating the experimental setup. Therefore, a successful reconstruction of the simulated test-lung data as shown in Table 1, in comparison to unaffected experimental breaths would demonstrate and quantify the method’s ability to reconstruct the ground truth from waveform data obtained in asynchronous breaths and provide confidence in the results using clinical data.

**Fig. 5:**
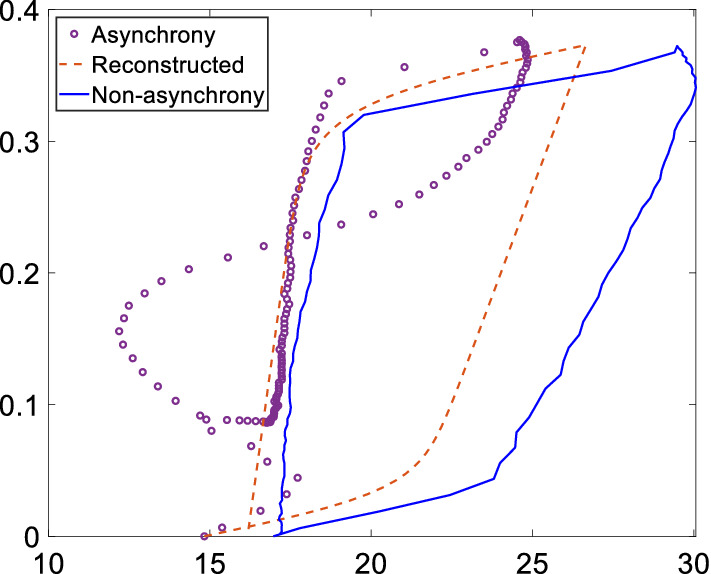
Example of inaccurate reconstruction due to missing *V*_*m*1_ during a very large asynchrony event beginning before the start of inspiration and severely altering waveforms and the PV loop

Using clinical data, 90% breaths were reconstructed with less than 10% RMS error. Larger errors are mainly due to the lack of necessary information for segment reconstruction as shown by example in Fig. 5, where turning point *Vm*_1_ is missing in the clinical data (circles) due to asynchrony beginning before the breathing cycle begin from the ventilator and this event having very large magnitude. This issue leads to estimation failure in finding *k*_1_ and *k*_2_ for the reconstruction. However, such heavily altered breaths are extremely rare in this clinical data set. Equally, the latter half of inspiration could potentially be used to estimate these values [39].

However, the occurrence of measurably inaccurate reconstruction is lower than 10%, thus only affecting the trend analysis of patient asynchrony. Clinically, the magnitude or impact of asynchrony might vary for each single breath, where, in contrast, patient condition may not vary for a short-period, such as 30 s or 60 s, equivalent to 10–20 breathing cycles. Thus, a moving average of reconstruction could be implemented across every 10–20 breaths (~ 1 min on average at typical breathing rates for ventilated ICU patients of 15–20 breaths per minute), enabling a steady and accurate reconstruction of ventilated response or fundamental mechanics, as well as the estimation of asynchrony severity in more clinically relevant periods, as the main treatment is to adjust MV settings in response to persistent asynchrony [40].

As seen in Fig. 5, the reconstruction errors mainly include HLA errors for parameter identification and HLM modeling error for reconstruction. Both errors affect the performance of reconstruction, while the HLM modeling errors are the cumulation of both HLA errors and model deficiency errors. Thus, reducing errors during HLA parameter identification would decrease both errors in the final reconstruction. However, the ability to reduce HLA errors is highly reliant on the magnitude of asynchrony shown in Fig. 5. Therefore, future work to improve the HLA and HLM reconstruction for significant asynchrony cases could reduce some errors, although very large asynchronies, while rare, may remove too much information from measured pressure, flow waveforms to allow meaningful reconstruction.

Clinically, the errors are also an upper case estimate. Specifically, the clinical reconstruction is compared to unaltered breaths when the patient is fully paralyzed at the commencement of a RM. Breath to breath variability would thus increase errors. The overall errors in Figs. 3 and 4 are still quite low and should be suitable for clinical studies to validate their clinical impact.

In addition, the proposed method provides a breath-to-breath real-time reconstruction of asynchrony as HLA and HLM identification and simulation are direct calculations suffering no convergence issues or high complexity. Therefore, steady and accurate reconstruction can be readily available in real-time via moving average of the current breath and previous setting breaths. These outcomes would be critical for practical clinical use.

Clinically, controlling energy dissipation is considered as an efficient tool to adjust MV settings to minimize ventilator induced lung injury [41]. Thus, the metric *E*_*asyn*_ used for comparison of the energy dissipation of the reconstructed and measured breathing cycles is proposed to estimate and quantify asynchrony magnitude. As shown in Fig. 4, there is a clear difference of *E*_*asyn*_ between major asynchrony (Patient 1 and Patient 2) and minor asynchrony (Patient 3 and Patient 4). Although the detailed relation of *E*_*aysn*_ to clinical diagnosis and choice of ventilator setting remains to be investigated based on a larger cohort of patients, the identification of asynchrony occurrence alone provides a clinically useful alarm of poor MV setting or low sedation if, for example, *E*_*aysn*_ is greater than 10% for an extended clinically defined period. Finally, detection of large *E*_*aysn*_ also suggests a significant SB effort, which might assist a clinical decision to switch invasive MV to non-invasive MV.

Finally, research employing both simulated and clinical data for a proof-of-concept validation of asynchrony identification is quite limited. As the observed patterns of asynchrony from clinical data were successfully replicated using the test-lung experiment developed for this study, this study is able to provide a unique comparison and validation of the proposed method in both controlled and real scenarios, thus offering a relative more complete evaluation of the method. It is worth noting that numerical data generated from a mechanics-based computational model considering the coupled effect of passive response and patient effort would also offer a controlled scenario for validation equivalent to the test-lung data as they both provide known ground truth. Thus, the performance of the method against numerical data should be expected to be similar to the experimental validation in this study. Therefore, numerical data would be a good option for validation particularly if test-lung data are not available.

The overall results show the potential of the virtual patient model and its feasibility for different asynchrony types from practical clinical monitoring perspective. However, its robustness and full generalizability requires a larger cohort of clinical data with different patient conditions and lung mechanics. In addition, a further improvement for clinical use might be achieved by pre-defining a wider range of typical asynchrony patterns for different MV modes, thus enabling more efficient and accurate reconstruction, and classification, with additional constraints.

## Conclusions

This study extends a clinically validated virtual patient model from fully ventilated respiratory response to asynchronous breathing cycles without undermining its ability to be automated or its calculation efficiency. Identification and reconstruction are validated against both test-lung experimental data with known ground truth and real clinical data. Results show the accuracy and robustness of the method for the reconstruction of unaltered PV loops capturing the true underlying mechanics. More importantly, the modeling and reconstruction can be implemented breath-to-breath in real-time, which is critical for practical clinical use. In addition, an energy-dissipation metric *E*_*asyn*_ is proposed and validated to evaluate asynchrony magnitude, from which larger cohorts could be used to devise thresholds for clinical decisions based on asynchrony magnitude. Finally, the versatility of the proposed virtual patient model for several typical MV cases is also validated, where its ability to be automated breath-to-breath enables realistic use for bedside monitoring and guiding MV care.

## Methods

### Hysteretic modeling of ventilated PV loop

The HLM lung mechanics model is described as follows [7]:

1 $$\ddot{V}+R\dot{V}+{K}_{e}V+{K}_{h1}{V}_{h1}+{K}_{h2}{V}_{h2}={f}_{V}\left(t\right)+PEEP,$$

where *V* is the volume of air delivered to the lungs widely used in respiratory mechanics models [6], and *K*_*e*_ represents the alveolar recruitment elastance. *V*_*h*1_ and *V*_*h*2_ are hysteretic volume response during inspiration and expiration, respectively, representing the key characteristics of nonlinear stress–strain or force–deformation relation, thus critical for determining the two nonlinear hysteretic springs, *K*_*h1*_ and *K*_*h2*_, for alveolar hysteresis elastance during inspiration and expiration, respectively. *R* is the airway resistance, PEEP is the positive end-expiratory pressure, and ${f}_{V}(t)$ is a steady-state input force.

For each breathing cycle, a hysteretic PV loop can be constructed using measured clinical pressure and volume (or flow) data from a ventilator. Figure 1 shows a typical constructed hysteresis loop without patient SB effort. To model the clinical PV loop using in Eq. (1), hysteresis loop analysis (HLA) is implemented as a first key step to identify the stiffness (*k*_1_-*k*_4_) and breakpoints (*V*_*m*1_, *V*_*m*2_, *V*_*m*ax_) of each nonlinear phase for a complete breath, yielding the dashed lines in Fig. 1 [38, 42]. Specifically, *V*_*m*1_ is the lower inflection point controlling the sudden increase of lung compliance during inspiration, *V*_*m*2_ is the upper inflection point defined for the sudden drop of compliance during expiration, and *V*_*m*ax_ is normally equal to the setting tidal volume. The stiffness parameters (*k*_1_-*k*_4_) are related to the alveolar recruitment elastance *K*_*e*_, alveolar hysteresis elastance during inspiration *K*_*h1*_ and expiration *K*_*h2*_, which are calculated as follows:

2 $${K}_{e}={k}_{2},$$

3 $${K}_{h1}={k}_{1}-{k}_{2},$$

4 $${K}_{h2}={k}_{3}-{k}_{4}.$$

**Fig. 6:**
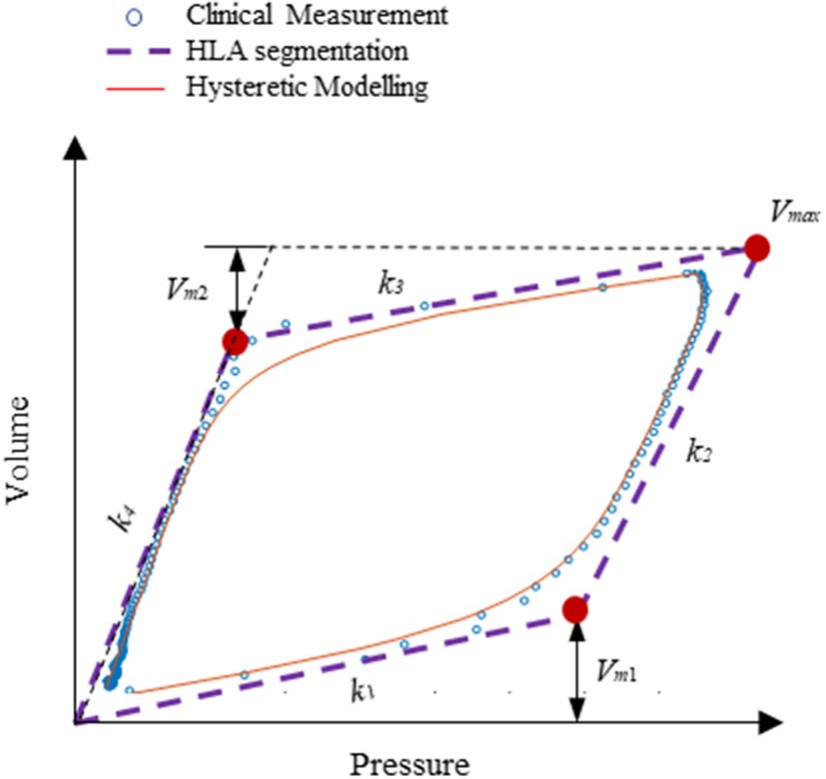
Example of HLM modeling of a fully controlled clinical PV loop

HLM model parameters can then be derived and identified to replicate the PV loop using Eq. (1), as seen by the solid red line in Fig. 6. A detailed derivation of the model parameters based on HLA segmentation in the HLM lung mechanics model can be found in [7], which is based in part on the prior basis function methods and models of [5, 8, 43].

### Asynchrony type and HLA identification of asynchronous PV loops

**Fig. 7:**
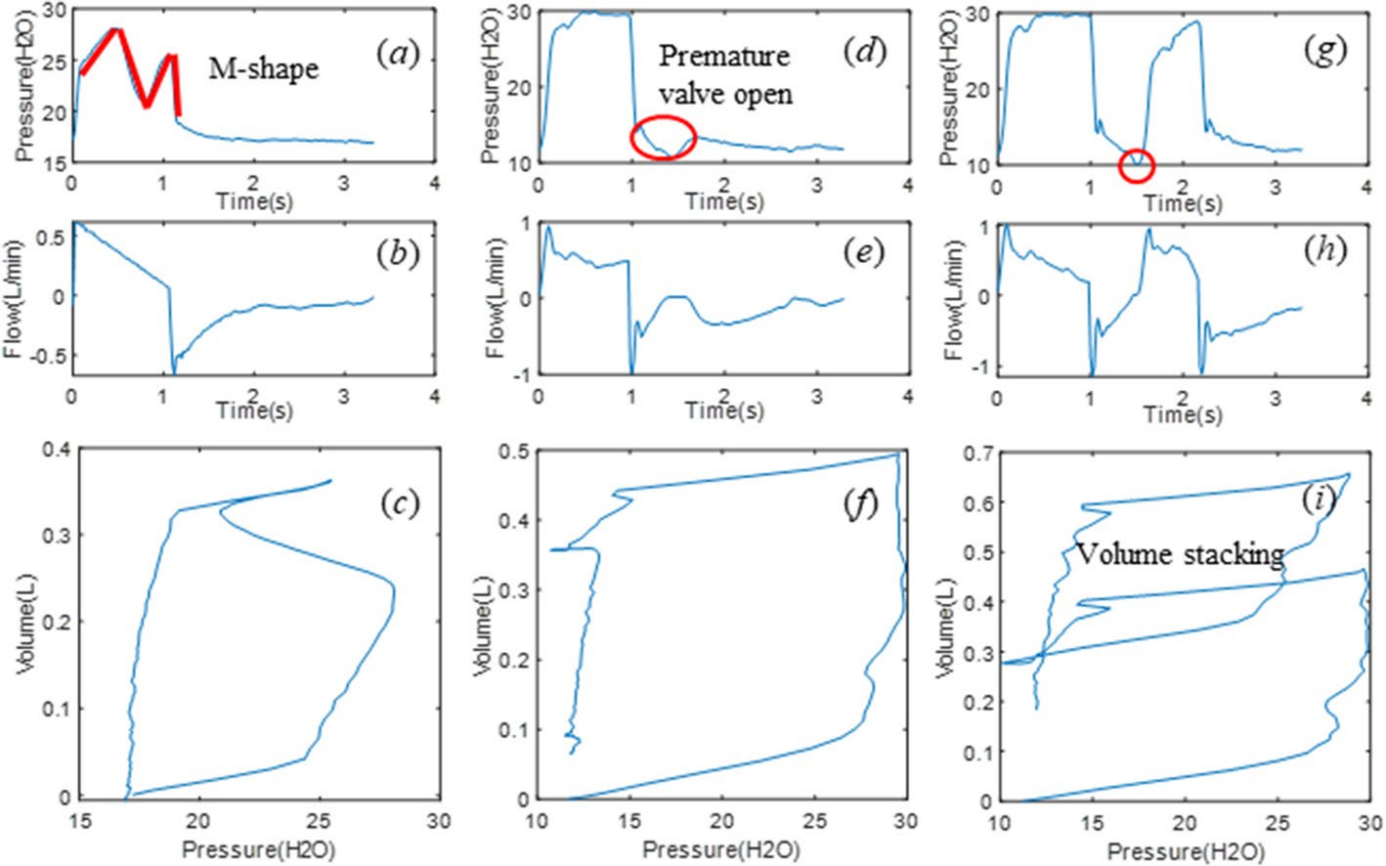
Three types of asynchronous breathing observed in both inspiration and expiration phase of clinical breathing cycles, with reverse triggering for the first column, premature cycling for the second column and double triggering for the third column. The first row shows the measured pressure, the second the measured flow, and the third the measured PV loop

Patient SB effort changes airway pressure and flow curves at any point in a breath, resulting different types of asynchrony. Reverse triggering is a common type of asynchrony due to a reflexive neural response triggered by the ventilator applied pressure and flow [26, 44], yielding the “M”-shaped pressure curve in Fig. 7a, where patient effort is triggered by ventilator-driven inspiration. In contrast, premature cycling occurs at the beginning of the expiratory half-cycle when the patient inspiratory time is longer than ventilator-defined inspiration, leading to a premature valve opening as seen in Fig. 7d. Premature cycling can also result in double triggering if patient effort exceeds the ventilator trigger threshold, activating a second breath and resulting volume stacking, as in Fig. 7g.

Therefore, patient asynchrony can occur during both inspiration and expiration, while the single compartment model is more focused on the modeling of inspiration, thus might be less effectively useful for premature cycling and double triggering [39]. Considering the measured PV loops in Fig. 7c, f, i, the HLA method would find more than the 2 lines for each of inspiration and expiration seen in Fig. 6, thus identifying the presence of asynchrony. Hence, in this study, HLA is employed to identify the presence, and thus incidence, of asynchrony. The identified parameters from HLA can then be used to calculate the model parameters for HLM, enabling reconstruction of an unmodified breath without asynchrony, which in turn enables calculation of the magnitude of any given identified asynchrony.

The overall method to identify and quantify asynchrony includes 3 major steps as follows:

#### Step 1: Identify and classify the asynchrony

**Fig. 8:**
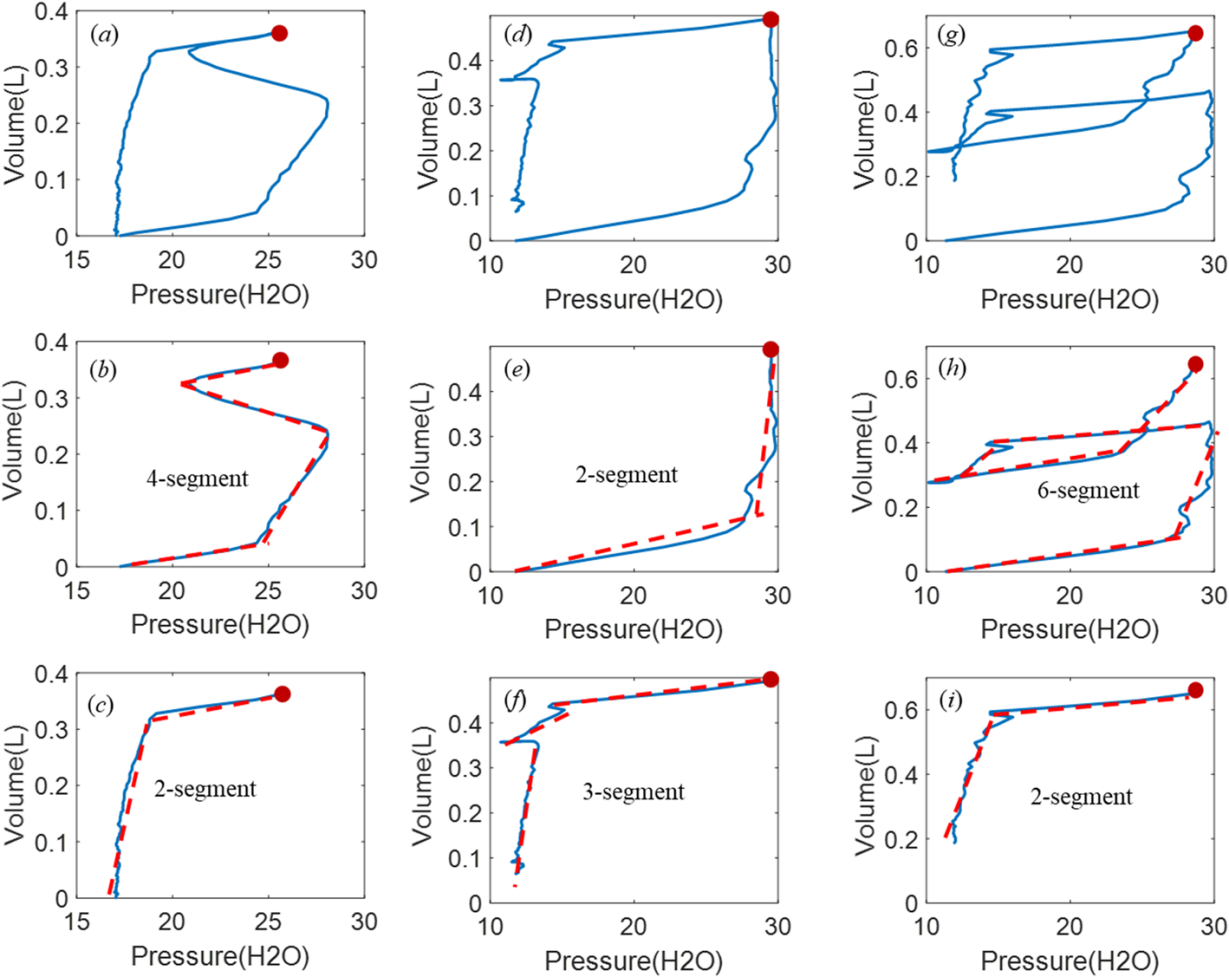
HLA segmentation of asynchronous PV loops showing the same PV loops as Fig. 2c, f, i in the first row, and the segments for inspiration in the second row up to the turning point (red dot) at peak volume before expiration segments in the third row

The reconstructed PV loop is divided into two phases using the turning points where the volume is maximum, as shown in Fig. 8 for the asynchronous PV loops of Fig. 7c, f, i. For each phase, the optimal number of segments is identified using HLA with the change of stiffness and breakpoints obtained for each segment. Thus, a 2-segment half-cycle indicates ventilated breathing response without SB effort, while anything more than 2 segments identifies and asynchrony and can uniquely classify the type of asynchrony. Specifically,

1. A 4-segment half-cycle during inspiration with stiffness changing from positive to negative and reversing to positive near the turning point indicates a reverse triggered asynchrony in the PV loop (Figs. 7c and 8a).
2. A premature cycling PV loop includes 3 segments in the flow waveform during expiration with the middle segment generated by the longer patient-driven inspiration, but a relatively unmodified pressure trajectory (Figs. 7f and 8d).
3. Double triggering induces a second breath half-cycle in the first phase of the PV loop, thus creating an extra 2 to 3 segments during inspiration, yielding 5 to 6 segments depending on the location of the double triggering during expiration (Figs. 7i and 8g).

#### Step 2: Identify the stiffness and breakpoints for the ventilated PV loop

**Fig. 9:**
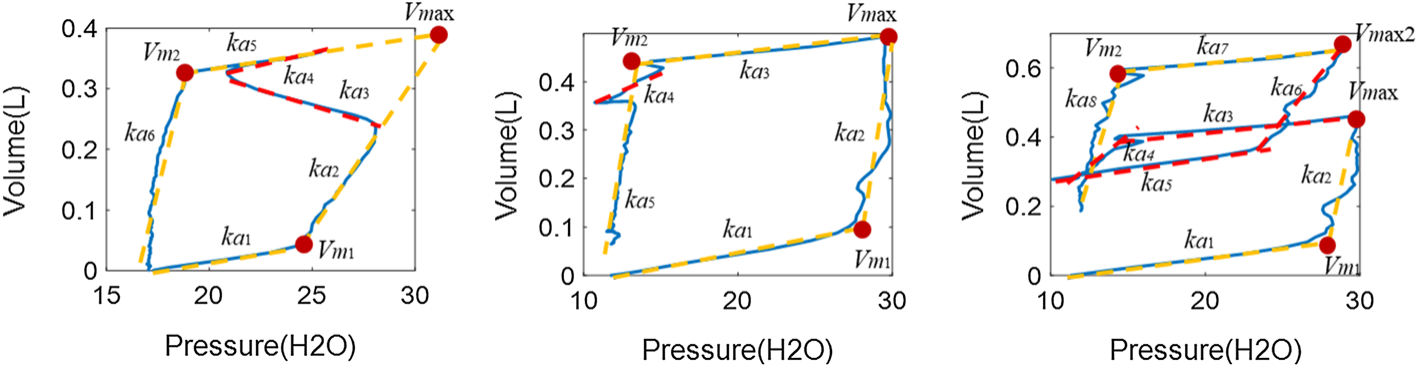
HLA identification of HLM stiffness and breakpoints

With the identified type of asynchrony from *Step* 1, the stiffness and breakpoints for the ventilated PV loop can be obtained using the HLA parameters, as shown in Fig. 9.

1. *Reverse Triggering* Stiffness values *k*_*a*1_–*k*_*a*6_ and breakpoints *V*_*m*1_–*V*_*m*2_ are identified for reverse triggering. The breakpoint *V*_*max*_ for fixing the tidal volume in the reconstructed breath can be calculated based on the intersection point of the *k*_*a*2_ and *k*_*a*5_ segments, while the stiffness parameters, *k*_1_, *k*_2_, *k*_3_ and *k*_4_, for the unaffected ventilated response are readily obtained from the identified *k*_*a*1_, *k*_*a*2_, *k*_*a*5_ and *k*_*a*6_ values, respectively.
2. *Premature Cycling* Stiffness *k*_*a*1_–*k*_*a*5_ and breakpoints *V*_*m*1_ and *V*_*max*_ are identified for premature cycling. The stiffness parameters *k*_1_, *k*_2_, *k*_3_ and *k*_4_ correspond to *k*_*a*1_, *k*_*a*2_, *k*_*a*3_ and *k*_*a*5_, respectively. The breakpoint *V*_*m*2_ can be calculated using the intersection point of *k*_*a*3_ and *k*_*a*5_ segments.
3. *Double Triggering* Stiffness *k*_*a*1_–*k*_*a*8_ and breakpoints *V*_*m*1_–*V*_*m*2_ and *V*_*max*_ are identified for double triggering. The stiffness parameters *k*_1_, *k*_2_, *k*_3_ and *k*_4_ correspond to *k*_*a*1_, *k*_*a*2_, *k*_*a*7_ and *k*_*a*8_, respectively. The breakpoint *V*_*m*2_ can be calculated using the intersection point of *k*_*a*3_ and *k*_*a*5_ segments. Note the breakpoint *V*_*m*2_ is calculated via *V*_*max*2_.

#### Step 3: Calculate the HLM parameters for forward simulation of ventilated response

**Fig. 10:**
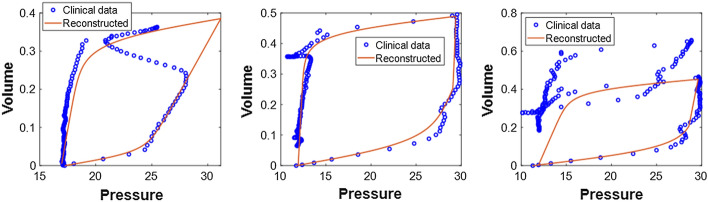
Model reconstructed PV loops from Fig. 4 using the identified HLM and segments related only to the underlying lung mechanics

The HLM includes ten (10) parameters for modeling the lung hysteresis mechanics, which can be directly calculated using the HLA identification results (*k*_1_–*k*_4_, *V*_*m*1_, *V*_*m*2_, *V*_*m*ax_), as detailed in [7]. Therefore, given the identified HLM model, the reconstructed and unmodified ventilated response and PV loop can be replicated using forward simulation, as shown in Fig. 10 for the same PV loops as in Figs. 7, 8, and 9. The entire process is directly identifiable by solving a convex problem, thus suffering no convergence or identifiability issues [45–49]. Hence, it provides a fast and robust reconstruction for the ventilated PV loops compared to other complex FEM and iteration methods.

### Estimation of asynchrony effect

Energy dissipation refers to the work done by the ventilator in the airway for a ventilated patient, and can be directly calculated from the enclosed area of a measured PV loop. It is a critical measure of energy required for ventilating a patient and thus represents the essential patient recruitability [41], where a high energy or work of breathing would indicate a stiff lung and a less recruitable patient. Patient asynchrony produces negative work against the work of breathing done by ventilator, reducing its total value. Hence, the difference of area between the asynchronous PV loop and the reconstructed PV loop indicates the magnitude of asynchrony relative to the ventilated energy required to support breathing, yielding a proposed metric defined as follows:

5 $${E}_{asyn}=\frac{{A}_{ventilated}-{A}_{asyn}}{{A}_{ventilated}}\times 100 \%,$$

where *E*_*asyn*_ is the quantified measure of the asynchrony effect in a breathing cycle, *A*_*ventilated*_ is the area of reconstructed ventilated PV loop without asynchrony, and *A*_*asyn*_ is the area of the asynchronous PV loop, where these differences are evident in Fig. 10.

**Fig. 11:**
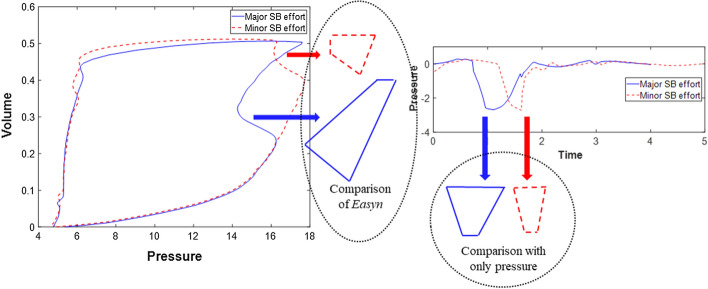
Schematic of *E*_*asyn*_ comparison for the estimation of asynchrony effect using the reverse triggered example of Fig. 2c

Therefore, *E*_*asyn*_ shows the difference of areas, and thus the work done, between the fully controlled breath and contaminated breath with SB effort. A larger *E*_*asyn*_ would indicate a more significant SB effort or asynchronous waveform superposition to the observed breathing cycles, as shown in Fig. 11. More importantly, both differences of pressure and volume are accounted for in calculating *E*_*asyn*_ to offer better insight into the change of work done based on physical definitions, where prior works used only changes in airway pressure for quantifying asynchrony magnitude [24], potentially misestimating the magnitude (Fig. 11). Therefore, the proposed metric *E*_*asyn*_ provides a different and more significant contrast of changes of SB effort based on energy dissipation.

**Fig. 12:**
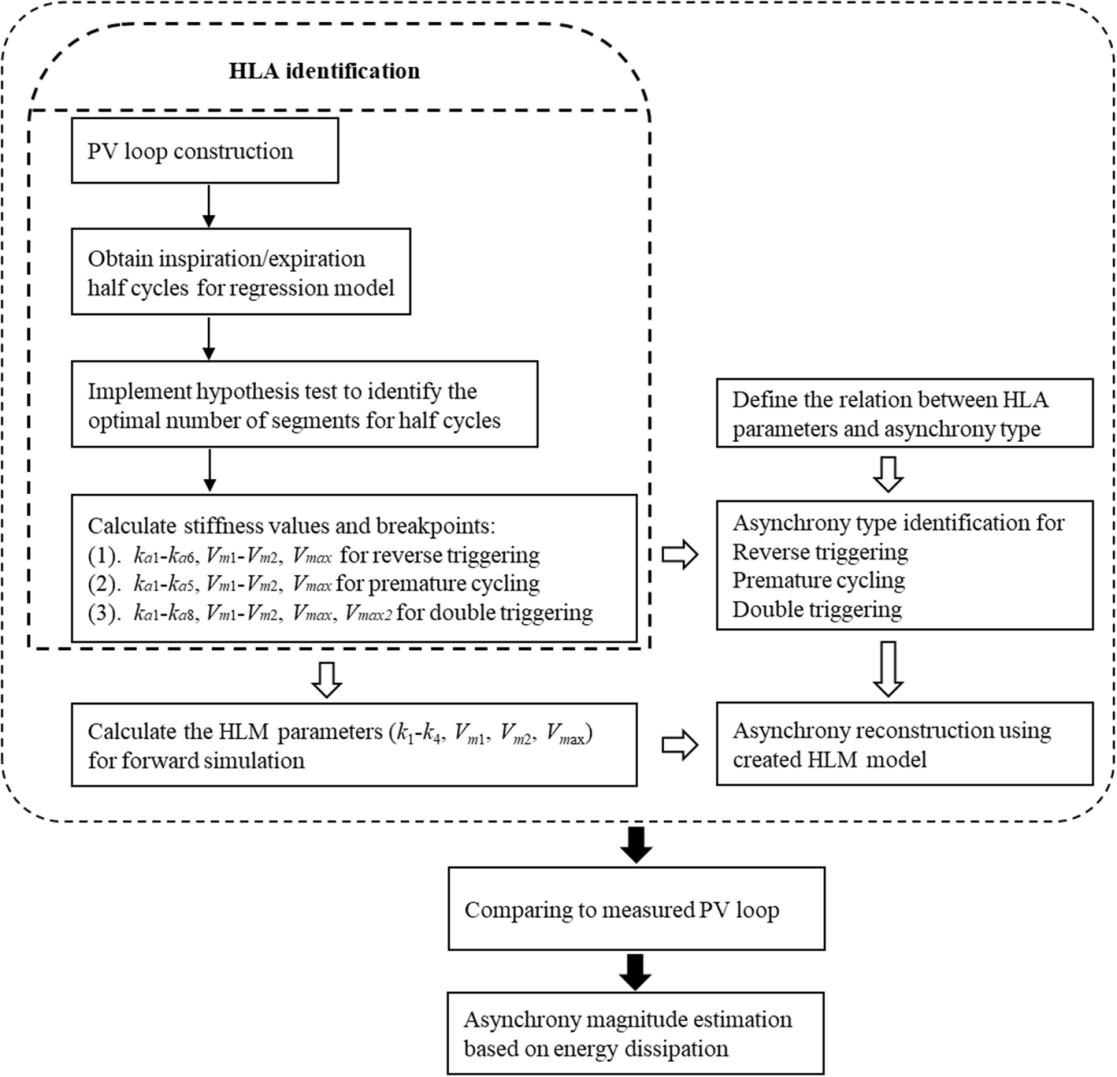
Flow chart for asynchrony reconstruction and estimation

Finally, Fig. 12 shows a flow chat for the implementation of the asynchrony reconstruction and estimation algorithm based on the proposed method.

### Laboratory test-lung data

The laboratory study first provides a proof-of-concept validation for asynchrony identification using experimental data generated from mechanical test lungs. In this case, the occurrence and type of asynchrony, as well as its true magnitude are known to enable accurate performance assessment. Asynchrony is simulated using two QuickLung test lungs (INGMAR Medical, USA) connected to a Mindray SV800 ventilator (Mindray, China). Reverse triggering is simulated in VC (volume control) and PC (pressure control) ventilation modes, and double triggering and premature cycling for PC mode only to match the observations from the clinical data used in this study.

**Fig. 13:**
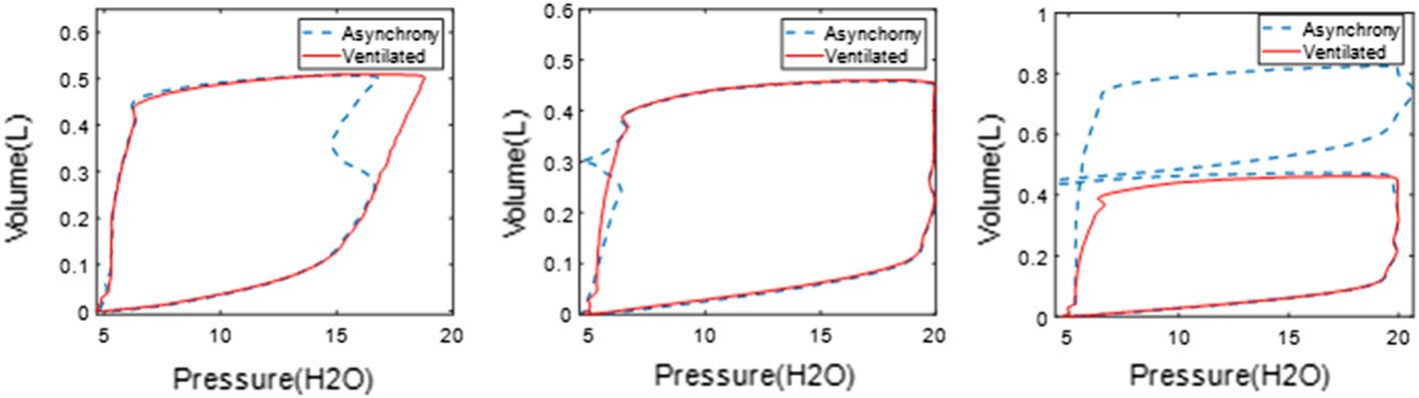
Typical experimentally simulated asynchronous breathing for each asynchrony type

Table 2 provides the details of description for the 3 simulated scenarios. Each scenario includes 10 ventilated breathing cycles and 10 asynchronous breathing cycles to compare and validate the accuracy of the reconstruction. The test-lung experiment setup thus also provides a known ground truth for a fully sedated and paralyzed breath, simulated without any asynchronous alteration of the breathing cycle. Figure 13 shows typical experimental normal and asynchronous PV loops for each scenario, which shows a similar pattern of asynchrony to the clinical PV loops in Figs. 7, 8, 9, and 10.

**Table 2:** Description of 3 simulated asynchrony scenario

Scenario	MV mode	Asynchrony type	Definition	Experimental protocol
1	VC	Reverse triggering	Mechanical inflation leads to a reflexive neural response, known as entrainment	Lifting the top panel of test lung to create a negative pressure during the inflation of test-lung bellow
2	PC	Premature cycling	Patient inspiration time is longer than mechanical inspiration	Hold the top panel of test lung to create a negative pressure and positive flow during the deflation of test-lung bellow
3	PC	Double triggering	Patient inspiration is longer than mechanical inspiration and patient effort exceeds the ventilator trigger threshold	Lift the top panel of test lung to create a negative pressure exceeding the trigger threshold during the deflation of test lung bellow

### Clinical data

Clinical data from 4 MV patients from a clinical pilot trial for the CURE trial [50] conducted in the Christchurch Hospital ICU are used for proof-of-concept validation of the proposed reconstruction method based on the HLM virtual patient model [51, 52]. Airway pressure and flow data were collected with a sampling rate of 50 Hz from a Puritan Bennett 840 ventilator (Covidien Boulder, CO, USA) [52]. The New Zealand Southern Regional Ethics Committee granted ethics approval for this pilot trial [51]. Patient demographics are given in Table 3. Asynchrony types with reverse triggering, premature cycling and double triggering were observed using manual inspection for each patient in Table 3, thus matching the simulated asynchrony scenarios using the test lungs.

**Table 3:** CURE patient demographics

Patient	Sex	Age	Diagnosis	MV mode	Asynchrony types
1	Female	53	Fecal peritonitis—surgery	VC	Major reverse triggering
2	Male	34	Trauma	PC	Major double triggering and premature cycling
3	Male	60	Pneumonia	PC	Minor reverse triggering
4	Male	88	Pneumonia	PC	None

PC: pressure control; VC: volume control

In addition, each patient was given muscle relaxants to suppress SB effort before a stepwise recruitment maneuver (RM) given as part of the CURE trial [50]. Therefore, fully ventilated and controlled breathing cycles with SB effort eliminated by sedation and paralysis were observed at the same positive end-expiratory pressure during the first step of these RMs, enabling a comparison to reconstructed waveforms for further clinical validation. The reconstruction is finally applied to 20 min breath cycles collected before the RM. Specifically, reconstruction accuracy is quantified by comparing the pressure waveform to the observed breathing cycles using root mean squared error, defined as follows:

6 $$RMS=\frac{\sqrt{\frac{1}{n}\sum_{i=1}^{n}{\left({P}_{i}-{\widehat{P}}_{i}\right)}^{2}}}{\frac{1}{n}\sum_{i=1}^{n}{P}_{i}}\times 100 \%,$$

where ${P}_{i}$ is the clinical pressure data, ${\widehat{P}}_{i}$ is the reconstructed pressure data, and *n* is the number of points for a breathing cycle. A low RMS error indicates an accurate reconstruction, ensuring an accurate estimation of the asynchrony using the metric *E*_*asyn*_ in Eq. (5).

## Data Availability

The datasets generated and analyzed during the current study are not publicly available for privacy reasons but anonymized data are available from the corresponding author on reasonable request.

## Abbreviations

MV: Mechanical ventilation
SB: Spontaneous breathing
PV: Pressure–volume
HLA: Hysteresis loop analysis
HLM: Hysteretic lung mechanics
ICU: Intensive care unit
VILI: Ventilator-induced lung injury
RMS: Root mean squared
CDF: Cumulative distribution function
PC: Pressure control
VC: Volume control
RM: Recruitment maneuver
PEEP: Positive end-expiratory pressure
